# The effects of 7-week participation in football on personal well-being among male asylum seekers in a UK hotel

**DOI:** 10.1016/j.heliyon.2024.e36992

**Published:** 2024-08-27

**Authors:** Chris McManus, Ben Jones, Mike Rogerson, Joshua Butson, Dominic Micklewright, Gavin Sandercock, Alison Swartz

**Affiliations:** a School of Sport, Rehabilitation & Exercise Sciences, University of Essex, Wivenhoe Park, Colchester, Essex, CO4 3SQ, United Kingdom; b School of Health and Social Care, University of Essex, Wivenhoe Park, Colchester, Essex, CO4 3SQ, United Kingdom

**Keywords:** Football, Personal well-being, Asylum seeker, Contingency accommodation

## Abstract

**Background:**

This study examines the impact of participating in a seven-week football programme on the personal well-being of male asylum seekers residing in contingency accommodation in the UK.

**Methods:**

This repeated measures study included a cohort of participants who engaged in weekly football sessions and completed a well-being questionnaire (ONS4) over a continuous seven-week period. Longitudinal linear regression analysis using generalized estimating equations (GEE) was used to assess the relationship between personal well-being and weekly minutes of football participation (M1). A second model incorporating the total number of non-football activity sessions in the past week as a covariate was also implemented (M2).

**Results:**

Of the 73 participants who completed the questionnaire, 23 responded twice or more across the study period and were subsequently included in the analysis. Results revealed that 2 h of football participation within 7 days significantly improved aspects of personal well-being when compared with no football participation, including improved life satisfaction, feeling life is worthwhile, and happiness. Both models demonstrated a shift from ‘low’ to ‘medium’ ONS4 categorical score. Anxiety levels remained unchanged regardless of participation and model.

**Conclusion:**

We provide important evidence on the potential benefits of football participation for promoting personal well-being among male asylum seekers residing in contingency accommodation.

## Highlights

• Football improves the well-being of male asylum seekers in contingency accommodation.
• Significant increases in life satisfaction, sense of worth, and happiness.
• Participants' well-being was significantly lower than the national average.
• Use of a nationwide survey establishes credibility and a benchmark for future research.

## 1 Introduction

Globally, 89.3 million people were forcibly displaced in 2021 due to violence, fear of persecution, and human rights violations, as reported by the United Nations High Commissioner for Refugees [1]. Asylum applications in the UK increased by 63 % in 2021 compared to the previous year, reaching the highest number in almost two decades [2]. Furthermore, the use of contingency accommodation, for asylum seekers in the UK has risen from 2,577 to approximately 49,493 from March 2020 to December 2022 [3]. Contingency accommodation is primarily hotels, often in non-residential areas with no allocated health, education, or exercise/sports services. Although these hotels are intended to act as shorter-term temporary residences while applicants await the outcome of asylum claims, these claims are reportedly taking between one to three years to be processed [4].

Refugee journeys are complex and nonlinear, but often include exposure to stress and danger associated with departure from one's home [5]. Many refugees' and asylum seekers' mobility was prompted by exposure to traumatic events, including armed conflict and persecution, which are significant risk factors for high rates of poor mental health [6]. Mental health, as defined by the World Health Organisation, pertains to an individual's cognitive, emotional, and social well-being, encapsulating their capacity to handle stress, work productively, and make decisions [7]. It encompasses a range of conditions and disorders affecting one's mood, thinking, and behaviour. Experiences before migration, during migration and in new locations all affect the mental health of refugees. Significant social determinants of poor mental health include uncertain income, poverty, challenges with housing, language skills and interpretation, as well as experiences with stigma and discrimination [8].

Furthermore, separation from family members, uncertainty about asylum status, and reliance on aid can exacerbate psychological trauma [9]. A meta-analysis found that depression and anxiety-related disorders are prevalent in refugee populations at a rate of 40–45 %, which is twice that of labour/economic migrants [10]. The prolonged uncertainty awaiting an asylum claim outcome and the process of settling in a new country can have a profound and often negative impact on mental health [11].

The World Health Organization's Global Action Plan (2019–2023) recognizes the need to promote the health of migrants and refugees and highlights the lack of access to healthcare services as a contributing factor to negative mental health outcomes [12]. To address this, the provision of local mental health and psychological support services is recommended for refugees and asylum seekers, with the aim of promoting mental health and preventing or treating mental disorders [13]. Despite this recommendation, refugees often underutilize these services, possibly due to a lack of awareness, and may seek treatment for mental health problems in hospitals more frequently than the reference population, without referral [14].

In comparison with the term ‘mental health’, ‘personal well-being’ is a broader, multifaceted concept relating to an individual's overall quality of life. It encompasses evaluative well-being (life satisfaction), hedonic well-being (feelings of happiness, sadness, anger, stress, and pain), and eudemonic well-being (a sense of purpose and meaning in life), reflecting both physical and psychological states, life experiences, and societal conditions [15]. While mental health is a component of personal well-being, the latter also integrates aspects such as physical health and social connections, emphasising a holistic view of an individual's life experience.

Participation in physical activity, such as sports, has been shown to contribute positively to various aspects of personal well-being. Studies have revealed that individuals engaged in physical activities report higher levels of life satisfaction and happiness compared to those less active. For example, access to sports facilities and subsequent participation in physical activity were associated with increased happiness and life satisfaction in the US [16], and club sports participants exhibited greater well-being and quality of life than those involved in other forms of physical activity [17]. This suggests the social aspects of club sports might contribute to enhanced life satisfaction, highlighting the importance of social connections in personal well-being.

The theory of Basic Psychological Needs Satisfaction (a sub-theory of self-determination theory) links physical activity with well-being. It suggests that greater fulfilment of three basic psychological needs (autonomy, competence, and relatedness) enhances human motivation and well-being. Offered physical activity allows individuals to experience autonomy in terms of their volitional participation and via autonomy-supportive settings/environments [18], can enhance self-perceived competence (skill development and mastery) [19], and promote relatedness to others via social interaction and a fostered sense of belonging and social support [20]. The relationship between physical activity and positive well-being outcomes, such as happiness and resilience is widely acknowledged in the general population [21], and indeed, physical activity can be an effective intervention in promoting mental health and treating psychological disorders [22]. Several systematic reviews have highlighted physical activity as effective in decreasing post-traumatic stress disorder (PTSD) and depressive symptoms in individuals diagnosed with post-traumatic stress disorder [[23], [24], [25]]. The high prevalence of poor mental health in refugee and asylum seeker populations highlights the need for effective physical activity interventions to promote mental health and well-being. In addition to the positive impact on individual mental health, physical activity, including participation in sports, has the potential to facilitate social integration [26] and address the psychological effects of trauma [27].

Engaging in physical activity can be seen as a key part of a holistic approach to mitigating poor mental health. A recent meta-analysis exploring the effects of physical activity on well-being in migrants without formal diagnoses of mental disorder found positive trends in improved self-efficacy, coping strategies, social inclusion, well-being, and behaviour outcomes were all associated with physical activity interventions [28]. A relationship between a higher participation rate in sports activities (football, volleyball, boxing, partner acrobatics) and lower anxiety symptoms was observed in 38 male refugees living in a Greek refugee camp following an eight-week exercise intervention [29]. Where physical activity interventions have been performed in countries of asylum on migrant populations with no formal diagnosis of mental disorder, improvements in mental health have been reported in countries of asylum for migrant populations after following physical activity interventions based on Bollywood dance [30], Tai Chi [31] and yoga exercises [32]. These investigations indicate that exercise interventions are both feasible and can be effective for asylum seekers.

The existing literature has established links between improved mental health and physical activity in refugee populations who are not awaiting the outcome of an asylum claim, nor living in contingency accommodation. For those living in contingency accommodation, access to sports facilities is limited geographically and in terms of cost. In this context, little is known about how sports participation shapes perceptions of well-being. Given the extended waiting times associated with asylum claims [4], and significant populations currently living in contingency accommodation we sought to explore how football participation could impact well-being and reduce poor mental health.

### 1.1 Objectives

Despite extensive literature linking physical activity with mental health, the experiences of male asylum seekers residing in UK hotels remain relatively unexplored. The primary objective of this study is to identify the effect of football games on the personal well-being of male asylum seekers residing in a UK hotel. This exploration is underpinned by the hypothesis that regular participation in football, with provided access to facilities, will enhance their overall well-being. A secondary objective is to assess the influence of other self-reported exercises, distinct from football, on their well-being, offering insights into the broader benefits or nuances of various physical activities for this group.

## 2 Methods

### 2.1 Study design

The study was a non-randomised, repeated measures, panel design with a longitudinal component, where the entire hotel population had the opportunity to participate in association football and complete well-being questionnaires on a weekly basis for a total of seven continuous weeks. A repeated measures design was chosen to track the relationship between participants' well-being and their weekly football engagement. This methodology allowed observation of intra-individual variations in well-being based on football involvement. Data collected at successive intervals provided a detailed exploration of this association. On the basis that participants would be residing at the hotel accommodation for an unknown time while awaiting their asylum claim, we decided it would be unethical to deny participants access to take up the opportunity to participate in a potentially beneficial activity, thereby ruling out a waiting-list control group. Therefore, we took a pragmatic approach to participant sampling, implementing a convenience sampling method, whereby all residents of the hotel accommodation had the opportunity to participate at any stage throughout the 7-weeks.

### 2.2 Procedures

The activity took place between February and April 2022 at temporary accommodation in the east of England (Colchester, Essex), in collaboration with the School of Sport, Rehabilitation and Exercises Sciences at the University of Essex, Colchester Borough Council, charity groups Community360 and Refugee Action Colchester, and the hotel manager. During a period of scoping prior to study implementation, five meetings took place between the institution and the groups listed above. In these meetings, football was identified as the preferred sport, a choice influenced by informal conversations between charity workers and many residents during the planning phase of the study. This facilitated the design of the study, including transport arrangements, scheduling of football sessions, optimal periods for data collection at the hotel residence, and addressing language/translation requirements. The decision to select football as the primary intervention activity was informed by the residents' expressed preferences, embodying a participant-centred approach that underscores the importance of cultural sensitivity and engagement [33]. This strategic choice is rooted in the recognition that aligning the intervention with the interests and cultural contexts of participants significantly enhances their intrinsic motivation to engage, thereby increasing the likelihood of sustained participation and ensuring the intervention's relevance. This approach not only respects the autonomy and preferences of the participants but also leverages evidence suggesting that preference-based physical activities can lead to better mental health outcomes [34], higher intervention satisfaction [35], and improved overall well-being [36]. Our methodological decision, therefore, reflects a deliberate and evidence-based strategy to maximize the intervention's impact on the health and well-being of the refugee population involved, that emphasizes engagement, cultural sensitivity, and participant empowerment. During the 7-week data collection period, hotel residents also had access to additional activities such as table tennis and a fitness suite, in addition to local sports clubs providing ad-hoc activities.

Sports facilities at the University were utilised for this project, where indoor football was available for participants to play together. As part of the project, a private bus was organised to transport participants between the temporary accommodation and the University, at no cost to the participants. These trips were provided twice a week, and each football session lasted a total duration of 1 h. Whilst facilities and equipment were provided to the participants, there was no set structure to these sessions with respect to format or intensity, however, a sports centre staff member supervised each session. All procedures were approved by the University of Essex ethics committee (Ethics approval number: ETH2122-0615; 17/01/22). All survey participants gave written informed consent to participate, having access to consent and participant information in Arabic, Albanian, French, Georgian, Kurdish Sorani, Nepali, Oromo, Persian, Russian, Tigrinya and Urdu.

### 2.3 Participants

All residents (*n* = 101) living in the temporary hotel accommodation had the opportunity to participate in the study that was advertised via posters placed throughout the hotel. Hotel staff also informed residents about this voluntary opportunity. An *a-priori* requirement for inclusion in the analysis was set at a minimum of two survey completions per participant to enable the assessment of the repeated effect of football participation. Of the 73 individual participants who completed the survey, six entries included incomplete or erroneous data. Of these, 41 only completed the survey on one occasion, and 23 participants completed the survey twice throughout the seven weeks. Table 1 reports the baseline characteristics of participants who completed the ONS4 survey on a minimum of two occasions (*n* = 23).

**Table 1:** The sociodemographic characteristics of the participants (*n* = 23).

Variable	
Age [range]	29 [18-51]
*Nationality* (*n* [%])	
Afghanistan	1 [4.3]
Angola	1 [4.3]
Ethiopia	3 [13.0]
Iran	3 [13.0]
Iraq	1 [4.3]
Libya	1 [4.3]
Morocco	2 [8.7]
Nepal	1 [4.3]
Pakistan	1 [4.3]
Republic of Congo	1 [4.3]
Somalia	1 [4.3]
Sudan	4 [17.4]
Syria	3 [13.0]
*Survey Language* (*n* [%])	
Arabic	11 [47.8]
English	7 [30.4]
French	1 [4.3]
Kurdish	1 [4.3]
Nepali	1 [4.3]
Persian	2 [8.7]

### 2.4 Measures

Participants were given the opportunity to complete the questionnaire every seven days, consistently on the same day of the week to minimise day-of-week effects on positive and negative moods [37]. Due to the temporary nature of the accommodation, a unique combination of participants completed the questionnaire each week. Each subsequent time a participant completed the questionnaire, it was designated as visit 2, visit 3, and so forth, to track the progression of their responses over time The questionnaire comprised two sections exploring 1) sporting engagement and 2) well-being. Questionnaires were available in Arabic, Albanian, French, Georgian, Kurdish Sorani, Nepali, Oromo, Persian, Russian, Tigrinya and Urdu. On days when charity partners and caseworkers were in the hotel foyer for meetings to support the participants, electronic tablets were made available to collect the survey responses. Collecting data in this way was easiest for participants who were already in the foyer for other meetings and could draw on translators who were already present if they needed assistance in completing the surveys. Questions about physical activity included the type and duration of activities completed in the previous seven days. The list of activities was devised in consultation with hotel staff and included football, outdoor walking, table tennis and badminton.

The personal well-being measure (ONS4) [38] was used to assess well-being. This measure consists of four questions that capture three components of well-being: evaluative, eudemonic, and affective experience. Feelings of happiness, life satisfaction, a sense of being worthwhile, and the reduction of anxiety are closely linked to the fulfilment of the three basic psychological needs proposed by Self-Determination Theory (SDT): autonomy, competence, and relatedness [39]. Autonomy to pursue activities that align with one's values and interests contributes to happiness, life satisfaction, and a sense of being worthwhile. Autonomy also plays a role in anxiety reduction by fostering a sense of control over one's life. Sense of competence is associated with increased happiness, life satisfaction, and a feeling of being worthwhile; and is linked to lower anxiety levels as individuals gain confidence in handling challenges. A positive sense of belonging (relatedness) contributes to happiness, life satisfaction, and a feeling of being worthwhile [40]. Relatedness also acts as a buffer against anxiety by providing emotional support and fostering a sense of community.

The ONS4 questions are designated National Statistics by the UK Statistics Authority [41], approved as a Government Statistical Service Harmonised Principle [42] and recommended as subjective measures from the National Well-being Programme capturing distinct aspects of personal well-being [43]. Furthermore, the ONS4 is recommended as a preferred measure in instances where time and access constraints are present [44]. The ONS4 is a four-item questionnaire comprised of four independent items; life satisfaction (‘Overall, how satisfied are you with your life nowadays?’); worthwhile (‘Overall, to what extent do you feel that the things you do in your life are worthwhile?’); happiness (‘Overall, how happy did you feel yesterday?’); and anxiety (‘On a scale of where 0 is not at all anxious and 10 is completely anxious, overall how anxious did you feel yesterday?’). Each item is answered on a scale from 0 to 10 where 0 is ‘’not at all’ and 10 is ‘completely”. Participants had the chance to ask questions and seek clarification during the questionnaire completion. However, whilst the ONS4 is not a fully validated measure [43], reliability, reflecting the measure's stability over time, was substantiated in a study where retest intervals averaged 26 days, yielding reliability coefficients for life satisfaction (r = 0.74), happiness (r = 0.57), anxiety (r = 0.45), and feelings of worthwhileness (r = 0.65). These figures indicate a dependable level of consistency for life satisfaction and worthwhileness. In contrast, the measures for happiness and anxiety, which prompt respondents to reflect on their feelings "yesterday", demonstrate a more dynamic nature [45]. Furthermore, the validity of the ONS4 in measuring personal well-being has been examined through its internal consistency and correlation with established well-being constructs. The positive well-being items within the ONS4, namely, life satisfaction, the sense of worthwhile, and happiness show significant correlations not only among themselves (r ≥ 0.7) but also with other established well-being measures, including the ‘Investigating Choice Experiments Capability Measure for Older people/Adults’ (ICECAP-O/A) (r ≥ 0.58) and the Warwick-Edinburgh Mental Well-being Scale (r ≥ 0.66) [46]. These findings indicate a level of internal coherence within the ONS4 measures and a degree of alignment with broader well-being scales.

#### 2.4.1 Statistical analysis

Demographic variables were reported using descriptive statistics to describe the sample. The relationship between personal well-being and weekly minutes of football participation over time was assessed using a longitudinal linear regression technique, generalized estimating equations (GEE) analysis. Generalized estimating equations (GEE) analysis is based on the generalized linear model's methodology [47]. The repeated measures design is compatible with the GEE analysis, chosen for its ability to handle the non-independence of repeated observations. In the present study, participants' unique identification was applied as the random intercept, the fixed factor was minutes of football, and the visit number was specified as the repeated within-subject variable. For the GEE analysis, minutes of football were organised as 0 or 120 for each visit. The visit number was adopted as Visit 1, 2, 3 and 4 (related, respectively to initial, second, third and final (last) contact with the participant). This results in a single regression coefficient (β) that incorporates between and within-subject correlations, making use of all data available [48]. The beta (β) coefficient represents the estimated change in the dependent variable (satisfaction, worthwhileness, happiness, and anxiety), for every one-unit increase in the independent variable (120 min of football participation). This coefficient signifies both the direction of the relationship, positive (enhancement in well-being variable) or negative (reduction in well-being variable) and quantifies its magnitude. An exchangeable correlation structure (where correlations between subsequent measurements are assumed to be the same) was assumed. A second GEE model was constructed, repeating the previous design, but also including the total number of non-football activity sessions in the past seven days as a covariate. Where a statistically significant result was observed (*p* < 0.05) in minutes of football, a post hoc test of multiple comparisons with Bonferroni correction was applied. Statistical analysis was performed with SPSS version 28.0 for Windows (IBM SPSS, Chicago, United States).

## 3 Results

The number of sporting activity sessions recorded during the 7-week data collection period is displayed in Table 2. Of the 23 participants completing the survey on two occasions or more, 159 activity sessions were recorded. Participation in the football sessions contributed towards the highest activity type (45 %), of which a total of 18 participants took part. Therefore, five participants also completed the survey on two or more occasions without participating in the football sessions. Walking and table tennis were the second and third most reported activities (15 and 9 % respectively). Table 3 displays the personal well-being of individuals, based on a maximum of four repeated survey responses per person, categorized by their duration of football participation.

**Table 2:** Activity sessions listed by sport reported during the 7-week period. Participants completing the survey on two or more occasions are only included.

Activity	Number of sessions	Number of participants
Badminton	11	5
Basketball	7	2
Cricket	2	1
Exercise Class/Bootcamp	13	3
Football	72	18
Table Tennis	14	3
Cycling	0	0
Dance	1	1
Golf	0	0
Gym	7	4
Martial Arts/Boxing/Wrestling	4	1
Netball	0	0
Rugby	0	0
Hockey	0	0
Running	4	2
Swimming	0	0
Tennis/Squash	0	0
Volleyball	0	0
Walking	24	6

**Table 3:** ONS-4 Personal well-being across a maximum of four repeated survey responses per individual, over a seven-week period by duration of football participation (mean ± SD).

	*n*	0 min	*n*	120 min
Life Satisfaction				
1st response	11	4.4 ± 3.8	12	5.3 ± 2.2
2nd response	9	4.1 ± 3.0	14	5.2 ± 2.5
3rd response	2	1.0 ± 0.0	7	4.6 ± 2.7
4th response	2	2.0 ± 1.4	3	5.0 ± 2.0
Life Worthwhile				
1st response	11	5.5 ± 3.5	12	5.1 ± 2.0
2nd response	9	4.3 ± 3.2	14	5.7 ± 2.6
3rd response	2	0.5 ± 0.7	7	6.6 ± 3.3
4th response	2	2.0 ± 1.4	3	5.7 ± 2.1
Happiness Yesterday				
1st response	11	4.7 ± 3.5	12	6.0 ± 2.3
2nd response	9	4.1 ± 3.1	14	4.1 ± 2.7
3rd response	2	1.0 ± 1.4	7	4.3 ± 2.7
4th response	2	2.0 ± 1.4	3	6.0 ± 1.7
Anxiety Yesterday				
1st response	11	4.5 ± 3.9	12	4.8 ± 2.0
2nd response	9	5.4 ± 3.4	14	3.9 ± 2.1
3rd response	2	0.5 ± 0.7	7	4.9 ± 3.2
4th response	2	2.0 ± 1.4	3	5.3 ± 0.6

The GEE analysis indicates the associations between participation in 120 min of football weekly and four dimensions of personal well-being, as presented in Table 4. Initial results from the first model reveal significant enhancements in life satisfaction, worthwhileness, and happiness (P < 0.05), indicating a meaningful improvement in these aspects of well-being attributable to football participation. These findings demonstrate not only statistical significance but also the practical importance of football in fostering enhanced well-being. Conversely, anxiety levels did not show significant changes, suggesting that football's influence on this aspect of well-being might not be as pronounced or may require further investigation to fully understand. Upon adjusting for the total number of non-football PA sessions in the second model, a refinement in our understanding emerges. The beta (β) values for life satisfaction, worthwhileness, and happiness increase, from 1.48, 1.69, and 1.16 respectively in the first model, to 1.51, 2.03, and 1.40 in the second, indicating a stronger relationship between football participation and these well-being measures when considering other physical activities. This suggests that football offers unique benefits above and beyond general physical activity, reinforcing its distinct value in enhancing life satisfaction, worthwhileness, and happiness. The estimated marginal means for life satisfaction, worthwhileness and happiness yesterday, indicate that following football participation, ONS4 threshold scores transition from ‘low’ (0–4) to ‘medium’ (5–6), regardless of the model. However, despite an increase in the β value for anxiety from 0.22 to 0.78, this relationship remains statistically non-significant, underscoring the complex interplay between physical activity and mental health dimensions. These nuanced findings highlight the specificity of football's impact on well-being, illustrating that its benefits extend when adjusting for participants' engagement in other forms of physical activity.

**Table 4:** Mean estimates (β), 95 % CI and *P* values calculated with generalized estimating equations, according to the relationship between the longitudinal development of ONS survey response and the completion of either zero or 120 min of football per week, over a period of seven weeks.

	Model 1 (Football)				Model 2 (Adjusted for non-football physical activity)					
	β	95 % CI	P value	EMM	QIC	β	95 % CI	P value	EMM	QIC
**Life Satisfaction**										
0 min				3.65 [2.31; 5.00]					3.63 [2.25; 5.02]	
120 min	1.48	0.93; 2.86	0.037	5.13 [4.29; 5.97]	436.2	1.51	0.08; 2.94	0.039	5.14 [4.31; 5.98]	441.0
PA						0.02	−0.41; 0.46	0.915		
**Life Worthwhile**										
0 min				4.07 [2.59; 5.56]					3.86 [2.31; 5.40]	
120 min	1.69	0.32; 3.05	0.015	5.76 [4.80; 6.72]	487.7	2.03	0.53; 3.53	0.008	5.88 [4.86; 6.91]	488.9
PA						0.30	−0.18; 0.79	0.217		
**Happiness Yesterday**										
0 min				3.82 [2.54; 5.11]					3.68 [2.30; 5.05]	
120 min	1.16	0.06; 2.27	0.04	4.99 [4.21; 5.77]	470.5	1.40	0.15; 2.66	0.029	5.01 [4.27; 5.88]	476.4
PA						0.16	−0.37; 0.69	0.560		
**Anxiety Yesterday**										
0 min				4.36 [2.93; 5.79]					4.02 [2.33; 5.59]	
120 min	0.22	−1.22; 1.66	0.764	4.58 [3.68; 5.48]	475.8	0.78	−0.97; 2.52	0.382	4.79 [3.88; 5.70]	438.8
PA						0.39	0.67; 0.72	0.018		

PA: non-football physical activity sessions. β: regression coefficient. 95 % CI: confidence intervals. EMM: estimated marginal mean. QIC: Quasi Likelihood under Independence Model Criterion.

## 4 Discussion

The study aimed to investigate the impact of football on the well-being of male asylum seekers residing in a UK hotel. The focus on those living in contingency accommodation awaiting asylum claims is novel in the UK context. Study participants provided with an opportunity to travel to and participate in association football twice a week over seven weeks were found to experience significant improvement in personal well-being, with increases in measures of life satisfaction, feeling that the things done in life are worthwhile, and happiness yesterday. This study's use of the ONS4, a well-established nationwide survey tool, enhances the credibility of our findings by providing a robust and standardised measure of personal well-being. The use of ONS4 allows for a meaningful comparison between the well-being of asylum seekers and that of the general population, thereby situating our results within a broader societal framework. This comparison not only validates the experiences of asylum seekers but also underscores the relevance of our findings for policy development and community integration efforts. Furthermore, employing the ONS4 enables systematic evaluation and facilitates the development of targeted well-being interventions for this demographic, ensuring that the specific needs of asylum seekers are addressed with precision.

Although participation in sports improved personal well-being, it is critical to note that current participants' average well-being scores were considerably worse than the UK average. Most recent data indicates that the UK report high levels (scores of 7–8) of life satisfaction, worthiness, happiness, and ‘low’ anxiety (scores of 2–3) [49]. Improving the mental health of asylum seekers is a key priority in the WHO Strategy and Action Plan for Refugee and Migrant Health [50]. Our findings are consistent with those of previous research, which has reported improved mental health following 12 weeks of self-selected physical activity (150 min/wk) in those resettled in a new host country [51]. Here we make an additional contribution to the literature by exploring the experiences of those awaiting asylum claim outcomes. This could be likened to the findings of a recent cross-sectional survey of asylum seekers residing in large housing facilities in Sweden, which reports that individuals with greater levels of physical activity are associated with less PTSD symptom severity [52].

The prevalence of mental health issues in the asylum seeker population indicates the importance of early intervention [53]. Furthermore, prolonged stays in contingency accommodation can exacerbate stress levels and heighten feelings of uncertainty, which are known to compound personal well-being challenges [54]. The current study is especially pertinent in this context, as it provides evidence of augmenting personal well-being via football participation in male asylum seekers. We demonstrate that during the period of awaiting an asylum claim, whilst residing in contingency accommodation, as little as two football sessions per week can contribute toward improved well-being. Broadly speaking, taking part in football practice within the community is known to positively impact mental health, increasing well-being and connectedness [55]. Such improvements in well-being can be attributed to multiple mechanisms. Participation in sports like football provides essential opportunities for social interaction, fostering connections crucial for mental health and a sense of belonging [56]. The structured nature of these activities offers routine and purpose, particularly beneficial in unstable or transitional life situations. Furthermore, the physical and mental challenges presented in sports help in building resilience and enhancing cognitive functions, thereby contributing significantly to an individual's psychological well-being [57]. In addition to the social and psychological benefits outlined, the physical benefits of football play a pivotal role in improving well-being through several key mechanisms. The high-intensity, intermittent nature of football, characterized by frequent sprints, tackles, and changes in direction, significantly enhances cardiovascular health and endurance. This is reflected in the elevated mean heart rate observed during small-sided games, often exceeding 80 % of the maximum heart rate, which contributes to improved cardiac function [58]. Furthermore, football participation leads to adaptations including lower fat mass, enhanced muscle strength and improved blood pressure [59]. Such physical enhancements not only bolster an individual's physical capabilities but also contribute to a reduction in the risk of chronic diseases and the improvement of metabolic health markers. Thus, this study highlights the significance of sports-based activity as a proactive approach to alleviating the mental strain associated with indeterminate waiting periods in temporary housing, while potentially also promoting physical fitness benefits, social integration and community engagement among asylum seekers.

In the context of the escalating global refugee situation, this study's findings highlight the urgent need for innovative, cost-effective mental health interventions. The high prevalence of mental health distress among refugees and asylum seekers calls for such initiatives. However, this potential can be realized most effectively through cross-sector collaboration. Universities, with their research prowess, and organizations like Community360, with their local community connections, can collaboratively optimize the implementation of these interventions. Other partnerships with governmental bodies, NGOs, healthcare providers, and football associations can further enhance the reach and impact of these initiatives. These collaborations can collectively form a multi-faceted approach to address the complex needs of asylum seekers. Furthermore, the project is aligned with the WHO Global Action Plan on Physical Activity 2018–2030 (*Action 3.5; development and implementation of physical activity programmes for vulnerable or marginalized populations*) [60], providing evidence of the positive impact of physical activity, specifically football participation, on the personal well-being of male asylum seekers. The results of this study demonstrate that providing accessible opportunities for physical activity could improve the personal well-being of asylum seekers, who are a vulnerable and marginalized population. The study also contributes to the growing body of literature on the potential benefits of physical activity for refugees and asylum seekers and highlights the need for further research in this area.

Anxiety is a multifaceted phenomenon that can be divided into state anxiety (a temporary condition in response to some perceived threat) and trait anxiety (a more stable general tendency to perceive situations as threatening) [61].

Although the ONS measure used provides only a superficial anxiety score, it is more aligned to state anxiety due to the referenced time period within the item. It seems reasonable to suggest in relation to the findings for this measure, that probable anxiety-inducing stressors associated with participants' circumstances, such as uncertainty around their asylum application and challenging conditions within contingency accommodation, are likely to have dominated their appraisals of this construct [62]. Additionally, although the measure appears aligned more with state anxiety, any influences of participants’ trait anxiety on answering this item would have been less responsive to short-term interventions like a seven-week football program [63]. While physical activity is known to alleviate symptoms of state anxiety, its impact on trait anxiety might not be immediate or significant within the timeframe of the study [64]. Together this suggests the need for a multifaceted approach that considers the interplay of trait anxiety, state anxiety, and situational factors in this population.

### 4.1 Limitations of the study

Although our study found a significant improvement in personal well-being among male asylum seekers when participating in a football, there are several limitations to consider. Firstly, we recognize that addressing the macro-structural factors that create unequal opportunities in sports and other areas of life requires more than any one program can accomplish on its own. These factors are complex and interconnected, and cannot be resolved through a single initiative [65]. Secondly, the absence of a traditional control group in our study design is a notable limitation. While our approach compared well-being outcomes between participants engaged in football (120 min per week) and those not involved (0 min per week), residing in the same contingency accommodation, it may not completely isolate the effect of football from other potential variables. Both groups had similar baseline access to social interaction within the accommodation, which partially addresses concerns about variations in social interaction opportunities. However, this design cannot definitively ascertain the causal relationship between football participation and well-being improvements. Thirdly, we were not aware of the participants' prior psychological status, including whether they had been diagnosed with a mental health issue, as this was not assessed. This lack of preliminary psychological profiling could influence the interpretation of well-being improvements. Furthermore, the potential for self-selection bias, primarily driven by participants' pre-existing interest in football, is identified as a limitation that could affect the generalisability of our findings to broader populations. This form of bias is crucial to acknowledge, as it highlights the participants' inclination towards the intervention, which may not be representative of the wider refugee population who might benefit from physical activity interventions. Consequently, the observed benefits of football participation on the well-being of our participants may not be entirely attributable to the intervention itself but may also reflect the characteristics of individuals predisposed to engage in this sport. This nuance suggests that while our study provides valuable insights into the potential benefits of engaging refugees in preferred activities for enhancing well-being, the results should be interpreted with caution. Previous research has proposed various strategies to mitigate such biases, particularly in studies involving refugee populations. For instance, offering incentives to participants upon each interaction or assessment completion as a means to attract a more diverse cohort, including those who are less physically active or less intrinsically motivated towards the specific activity under investigation [29]. By adopting such measures, subsequent research can aim to provide more representative insights, thereby extending the applicability and impact of findings across various demographic groups. A final potential limitation was that we did not measure basic psychological needs in order to better-evidence the role of satisfying these needs, towards enhanced well-being. This omission was to minimise participant burden, given the extent of existing evidence of the link between PA participation and well-being/life satisfaction. Despite these limitations, our study provides important evidence of the potential benefits of physical activity, such as football, for promoting personal well-being among male asylum seekers residing in contingency accommodation.

### 4.2 Future research directions

This study provides valuable insights into the potential of football participation to enhance the personal well-being of male asylum seekers in contingency accommodation. However, several areas warrant further exploration to build on these findings and advance the field. Future research should consider extending the intervention period beyond seven weeks to better understand the long-term effects of regular physical activity on well-being. Prolonged participation may reveal more sustained improvements and potentially uncover delayed effects on psychological aspects such as anxiety, which remained unchanged in this study as well as broader health-related quality of life measures [66].

There is also a need to explore the comparative effectiveness of different physical activities. By examining the impact of team sports versus individual exercises, researchers can better understand how various forms of physical activity meet the diverse preferences and cultural contexts of asylum seekers, thereby optimising intervention strategies. Additionally, broadening the demographic scope to include participants of varying genders, ages, and cultural backgrounds will enhance the generalisability of findings and provide critical insights into how different subgroups respond to these interventions.

Incorporating more detailed psychological assessments could further enrich our understanding of the mechanisms through which physical activity influences well-being. Baseline mental health evaluations and measures of psychological need satisfaction would offer a deeper perspective on how autonomy, competence, and relatedness contribute to improved well-being [67]. Moreover, given the importance of social integration, future studies should examine the role of sports in fostering cross-cultural cohesion among asylum seekers and host communities, providing empirical support for the use of sports-based interventions to promote inclusivity and reduce anti-migrant sentiment [68].

## 5 Conclusion

In conclusion, this study demonstrates that engaging in 1-h football sessions twice a week can significantly enhance the personal well-being of male asylum seekers living in contingency accommodation in the UK. The study adds to the growing body of literature highlighting the potential mental health benefits of physical activity interventions for refugees and asylum seekers and underscores the importance of providing accessible opportunities for physical activity for this population. The results of this study have important implications for mental health professionals, sports organizations, and policymakers involved in supporting the personal well-being and social integration of refugees and asylum seekers. Further research is needed to build on these findings and develop more comprehensive interventions that can address the broader social and structural determinants of health and well-being for this vulnerable population.

## Funding

This study was funded by a grant from 10.13039/100014013UK Research and Innovation (RCP 15167) via the 10.13039/100010046University of Essex.

## Ethics declarations

This study was reviewed and approved by University of Essex Ethics Committee, with the approval number: ETH2122-0615. All participants provided informed consent to participate in the study.

## Data availability

Data will be made available on request.

## CRediT authorship contribution statement

**Chris McManus:** Writing – review & editing, Writing – original draft, Project administration, Methodology, Investigation, Funding acquisition, Formal analysis, Conceptualization. **Ben Jones:** Writing – review & editing, Methodology, Funding acquisition, Conceptualization. **Mike Rogerson:** Writing – review & editing, Methodology, Funding acquisition, Conceptualization. **Joshua Butson:** Writing – original draft, Investigation. **Dominic Micklewright:** Writing – review & editing. **Gavin Sandercock:** Methodology, Formal analysis. **Alison Swartz:** Writing – review & editing.

## Declaration of competing interest

The authors declare the following financial interests/personal relationships which may be considered as potential competing interests:Dr Chris McManus reports financial support was provided by 10.13039/100014013UK Research and Innovation.
